# Impact of a radiological protection campaign in emergency paediatric radiology: a multicentric observational study in Brazil

**DOI:** 10.1186/s13244-022-01180-0

**Published:** 2022-03-07

**Authors:** Mônica Oliveira Bernardo, Flávio Morgado, Alair Augusto Sarmet Moreira Damas dos Santos, Shane Foley, Graciano Paulo, Fernando Antônio de Almeida

**Affiliations:** 1 Pontifícia Universidade Católica de São Paulo, Sorocaba, São Paulo Brasil; 2 Universidade Federal Fluminense, Niterói, Rio de Janeiro Brasil; 3 Radiography and Diagnostic Imaging, University College Dublin, Belfield, Ireland; 4 IPC-ESTeSC, Coimbra Health School, Coimbra, Portugal

**Keywords:** Paediatric radiology, Radiation protection, Computed tomography, Radiography, Patient safety

## Abstract

**Purpose:**

To evaluate the impact of a paediatric radiological protection campaign, implemented in the emergency units of a healthcare provider network in Brazil. This campaign aimed to promote awareness among emergency department physicians, regarding justification of paediatric X-ray referrals for paranasal sinus, chest and CT exams, as a strategy to reduce exposure to ionising radiation.

**Method:**

Frequency analysis of common paediatric imaging referrals from 19 emergency departments was performed for a 3-year period (2015–2018) to coincide with before, during and after the implementation of the radiation protection campaign. The campaign was multifaceted and involved dissemination of educational materials and imaging referral guidelines along with quarterly meetings with participating centres' leaderships. Additionally, patient dose cards were distributed to patients/carers. The Chi-Square test was used to examine the association between the type of examination and the patient's age group. Exact-Fisher test was performed to check for an association between participant engagement and the existence of the radiation protection committee.

**Results:**

Referrals reduced by 25% following the campaign with no reports of misdiagnosis. Many referrals in the youngest age groups. In 15 units, a radiological protection committee was created to raise awareness and to create a multi professional team to communicate the risks and benefits of radiological procedure in children.

**Conclusion:**

The campaign resulted in a substantial reduction in radiological referrals while promoting a radiation protection culture. Simple education initiatives can contribute to savings in both finances and radiation doses, particularly important in radiosensitive cohorts.

## Key points

- Educational strategies promote appropriate use of radiological exams.
- Individual patient dose cards are a tool to control children's exposure.
- Multiprofessional and parents’ awareness contribute to limiting unjustified radiological exams.

## Background

The progressive increase in radiological referrals in recent decades, especially in computed tomography (CT), has been sizable, likewise in the paediatric population, mainly due to easy access, with high accuracy and quicker diagnosis, avoiding more invasive procedures [1, 2]. More than 10% of CT scans in the world are performed on individuals younger than 18 years old [3]. The National Council on Radiation Protection and Measurement reported a 20% dose decrease in medical radiation exposure of the US population from 2006 to 2016, which was attributed to changes in technology and implementation of a dose awareness campaign [4], yet CT remains the main contributor to population dose from radiological exams. Justification is the key radiation protection principle which aims to ensure benefits from radiological imaging examinations exceed any potential risks, yet publications repeatedly show low rates for both adult and paediatric cohorts [5–7]. The International Atomic Energy Agency (IAEA) [8] and World Health Organization (WHO) [3] have highlighted concerns with the effect of ionising radiation on children and adolescents due to their increased risk. These institutions have encouraged countries to promote radiation protection actions. In 2017, the IAEA promoted recommendations through the Bonn Call for Action, emphasising 10 actions to strengthen the radiological protection of patients, among them: improve implementation of justification; strengthen radiation protection education and training of health professionals; improve radiation benefit/risk dialogue; reinforce clinical audit; and also, to use information technology solutions as a tool to support clinical decisions available in the emergency care sector [9].

International campaigns on radiological protection [10–13] have promoted guidelines on appropriate indications of radiological exams in different clinical procedures and quality evidence-based referral guidelines are in existence [4, 14, 15]. Radiation protection campaigns have specific goals to help families make informed decisions about imaging exam appropriateness and the need to child-size the radiation dose [10].

This paper aims to raise awareness amongst healthcare professionals and service users about excessive exposure due to radiological exams. Radiation protection education strategies were implemented and a radioprotection campaign of the reasonable use of radiological examinations, in a private healthcare-associated unit in Brazil, performed in children assisted in the emergency service was evaluated.

### Objective

To evaluate the impact of a radiation protection campaign in Brazil, in the reduction of referrals of imaging procedures in a paediatric population.

## Methods

### Study design and ethics

A retrospective and prospective quantitative multicentric longitudinal study in Brazil was performed in 46 voluntary units of a national healthcare system from June/2015 to May/2018 in different states of the country, recruited by convenience sampling. The study was approved by a university research ethics committee (Brazilian Certificate of Presentation of Ethical Appreciation number: 68956317.2.0000.5373) and each participating unit filled out an adhesion and consent form for their participation.

### Procedures

The authors, together with the operational and communication team of the national health care provider, developed resources including an individual dose card [10] with campaign information and documenting radiological procedures (name of the child, date, incidence and type of exam and facility name); written and audio-visual educational materials for health professionals and patients; campaign implementation guide and imaging referral recommendations [4, 10, 14, 15] and Brazilian Health Authority criteria [16].

An engagement strategy was developed to raise awareness about the risks and effects of using radiological exams not necessary for diagnosis, to provide guides for use in the campaign, to instruct leaders to be multipliers in their services, and to collect data, through periodic quarterly meetings in person or by videoconference. This involved a multi-professional team (paediatric doctors, receptionists, nursing staff, radiographers, and radiologists) in the process of clarifying the campaign to the caregivers (parents), reducing pressure from parents and defensive medicine practices, seeking to provide support to doctors to strengthen the principle of justification before the imaging referral and using evidence-based recommendations. Success stories and difficulties of the participants were discussed during the meetings, with feedback from the analyses. Also, facilities representatives regularly reported perceptions of both professionals and parents during the campaign. Videos were developed and made available in the reception of emergency departments, along with banners and brochures explaining the campaign which were additionally placed in doctors' offices. The individual patient dose cards [10] were filled out by radiographers, after personalised training. This was sent to parents/cares to bring to consultations and keep as a patient's imaging history.

The specific recommendations of the guidelines on the campaign's awareness tools were disseminated at meetings and by email, based on the health surveillance rules of the Brazilian Health Authority [16], international recommendations and initiatives [4, 10, 17–22], recommendations on the indication of sinus exams and traumatic brain injury of the Sao Paulo Paediatric Society [23, 24]. The responsible radiologist of the unit was oriented to review the protocols of exams: lowest dose to maintain the quality for the examination report; limiting the exposure area and avoiding repeated series; measure patient thickness for "child-size" technique; proper collimation and shielding [25, 26].

### Data collection

Each health care facility filled and sent, monthly, based on the unit's billing database, a standard spreadsheet with the number of radiological examinations and of attendance, performed at the paediatric emergency room, monthly data from the years before, during, and after the campaign and sent to the authors to analyse. Facilities were also asked to report any diagnostic errors arising from the rationalisation of radiological examinations. One data system analyst centralised the data collection.

The collected data exams were chosen based on the most frequent radiographic exams (X-ray of paranasal sinus and chest) and all paediatric CT exams which were mostly brain with a minor percentage being abdominal and spine CT.

### Data analysis

The data were analysed using Stata Package version 16 (StataCorp, Texas, USA). The confidence level was 95%. The variable chosen for analysis was the percentage of radiological exams requested by attendances, in three periods: June 2015–May/2016 (before the campaign); June/2016–May/2017 (during the campaign) and June/2017–May/2018 (after the end of the campaign). The Shapiro–Wilk test for normality was applied and the data of the index of exams requested/attendances were not normally distributed. The Chi-Square test was used to examine the association between the type of examination and the patient's age group. Exact-Fisher test was performed to check for an association between the participant engagement and the existence of the radiation protection committee. Wilcoxon paired tests were done with the index of referrals/attendances before and after the campaign. Incomplete data was omitted from analysis.

## Results

**Fig. 1:**
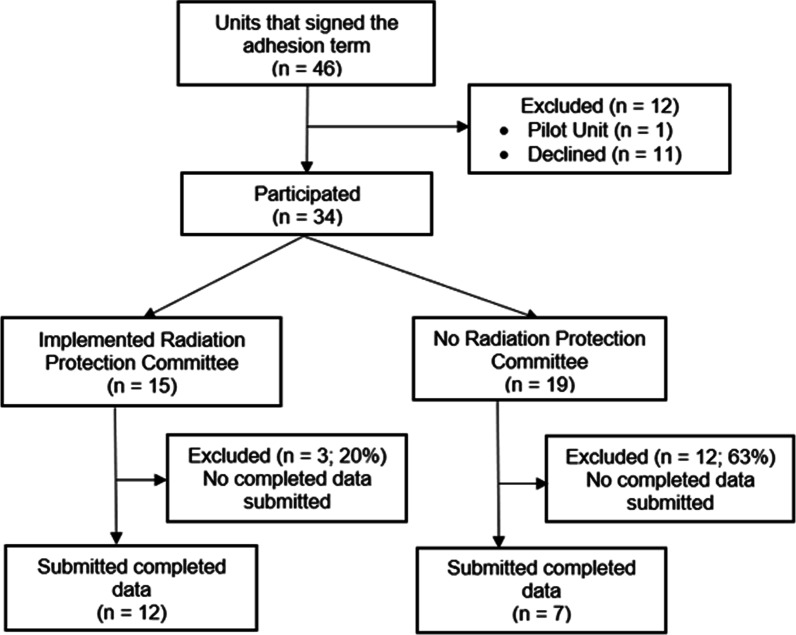
Campaign flowchart

Out of 123 facilities that attended the annual national healthcare system meeting, 46 units initially volunteered to take part in the project, 12 were excluded (11 later declined due to lack of resources and one was a pilot unit, which we decided not to include (Fig. 1).

Out of 34 units that participated, 15 (44%) have implemented a radiation protection committee, 12 (80%) of which submitted complete data. The Exact-Fisher test showed the significant association of participant engagement and the existence of a radiation protection committee (*p* = 0.0282). Units that did not submit complete data also reported a lack of resources.

This private health system studied has 29% of the national market share, 18 million beneficiaries, concentrated in the southeast and south region (86%), where the 19 participating units come from [27, 28], having 50,000–250,000 users, average per unit of 20,000–60,000 attendances per year.

**Fig. 2:**
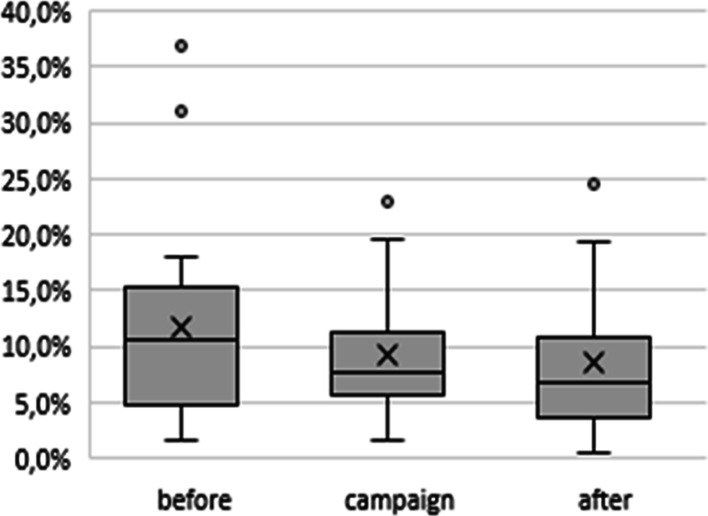
Exams requested/attendance index, before, during and after the campaign

The ratio of the number of radiological exams per attendance performed in each unit was evaluated (Fig. 2). Plotting data for the 19 participating healthcare facilities that sent complete data in the periods before, during and after the campaign, showed a reduction in the referrals for radiological exams/assistance.

**Fig. 3:**
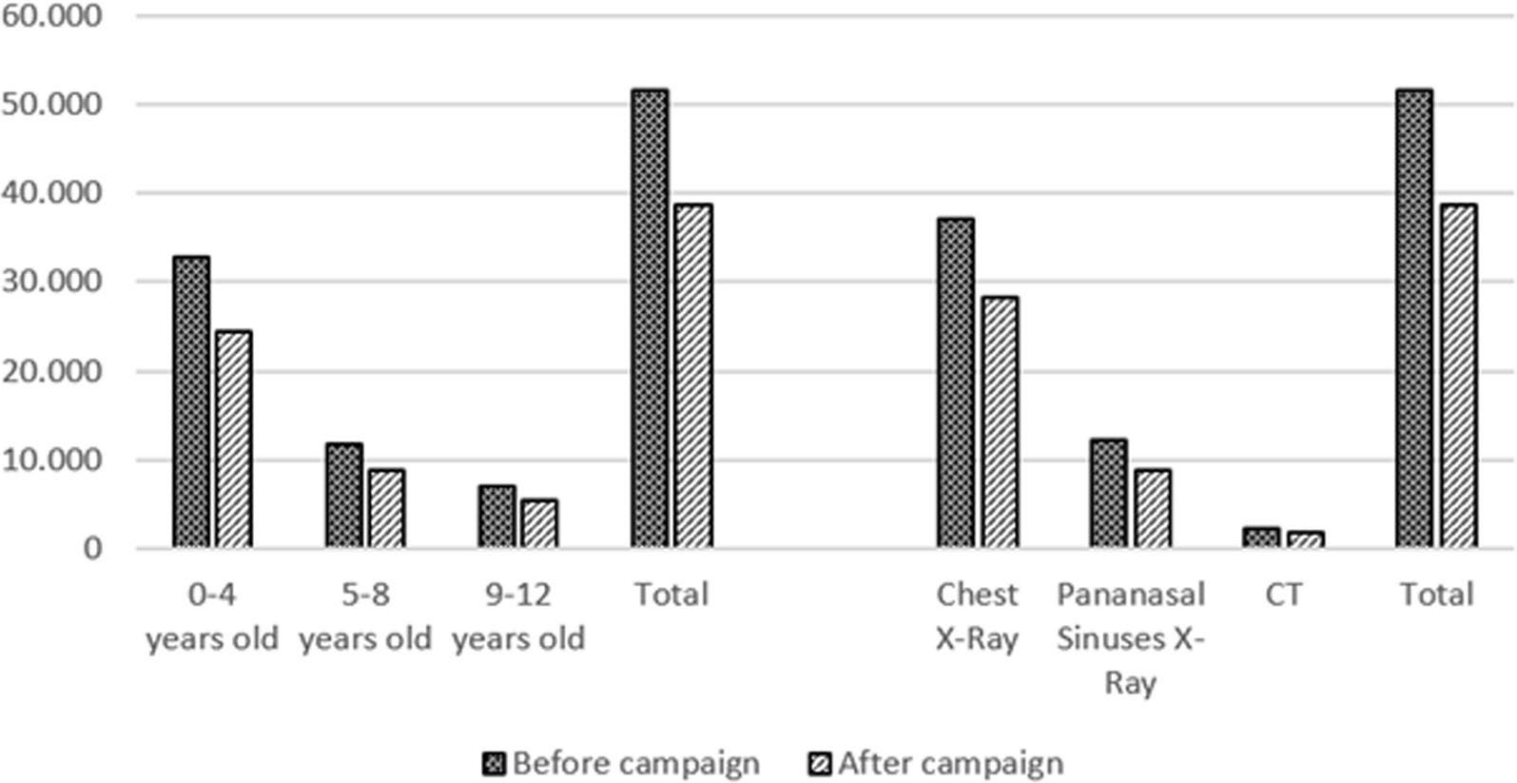
Exams before and after the campaign by age group and type of exam

In the 12 months after the campaign, there was a reduction in the radiological exams at paediatric emergencies in the engaged units (Fig. 3).

These reductions represented an overall reduction of 12,906 (25%) in the number of referrals for radiological exams in the attendances performed, after justification and implementation of the radioprotection campaign (Table 1).

**Table 1:** Facilities referrals/attendance and corresponding impact in the number of exams after the campaign

Facility	Referrals/attendance before campaign (%)	Referrals after campaign	Attendances after campaign	Referrals/attendance after campaign (%)	Exam reduction
A	7.67	295	5.083	5.80	− 95
	36.77	13.920	56.696	24.55	− 6.926
C	5.57	1.584	23.316	6.79	285
D	3.06	18	724	2.49	− 4
E	1.58	46	8.187	0.56	− 84
F	10.56	4.216	62.506	6.74	− 2.386
G	6.56	129	1.298	9.94	44
H	18.10	4.956	25.746	19.25	297
I	13.43	990	14.467	6.84	− 953
J	14.59	3.919	33.029	11.87	− 901
K	18.05	144	2.590	5.56	− 323
L	15.19	1.213	11.135	10.89	− 478
M	4.73	400	11.124	3.60	− 126
N	31.13	1.675	9.274	18.06	− 1.212
O	11.25	501	6.690	7.49	− 252
P	11.49	588	6.485	9.07	− 157
Q	3.13	103	5.256	1.96	− 61
R	7.11	3.977	49.608	8.02	448
S	2.75	64	3.096	2.07	− 21
		38.738	336.310		− 12.906
					− 25%

*The exam reduction is the difference between the referrals/attendance ratio before and after the campaign multiplied by the number of attendances after the campaign

**The overall reduction of 25% is the number of exams after the campaign (38,738) divided by the number of exams that would be done without the campaign (38,738 + 12,906). The reduction by type of exam and age group are shown in Table 2

**Table 2:** Impact after the campaign by type of exam and age group (Reduction of exams)

	0–4 years old		5–8 years old		9–12 years old		Total	
Chest X-ray	− 6.541	53%	− 1.541	13%	− 822	7%	− 8.904	73%
Paranasal Sinuses X-ray	− 1.638	8%	− 1.186	9%	− 693	6%	− 3.516	23%
CT	− 319	2%	− 291	1%	+ 124	1%	− 486	4%
	− 8.498	63%	− 3.017	23%	− 1.391	14%	− 12.906	100%

The Wilcoxon paired test was done with the index of referrals/attendance before and after the campaign of each healthcare facility and showed a significant percentage reduction of exams (25%) (*p* = 0.0065).

The largest number of exams performed in the institutions evaluated was Chest X-ray (73%), and the largest age group requested was 0–4 years old (63%). The Chi-Square test showed this significant association between the type of exam and the patient's age group (*p* < 0.01).

**Fig. 4:**
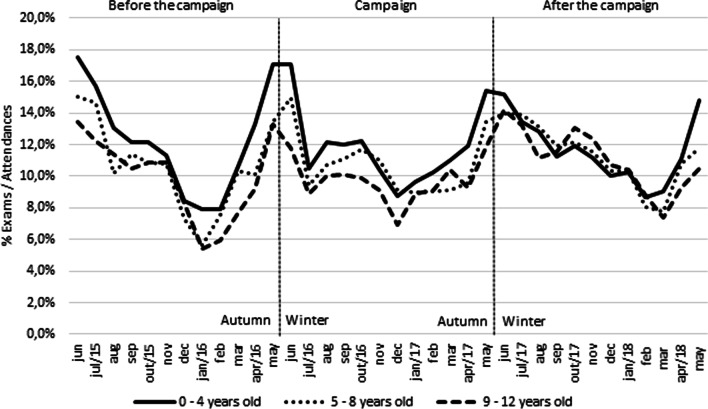
Monthly average exams requested/attendances by age group

The monthly graphical analysis showed an increase in the number of exams of Chest X-ray and Paranasal Sinuses X-ray requested between May and July (wintertime in Brazil), and between September and November (Fig. 4). The seasonality of the request for exams was observed in all units.

## Discussion

Of the 19 units that actively participated, there was a 25% reduction in the total rate of referrals for radiological exams in the emergency department post-campaign. The project corroborates the worldwide concern in encouraging educational activities and actions for radiological protection as well as guideline implementation. The paediatric radiological protection campaign is practical and effective and needs to be maintained with continuing education. The radiological protection committee created by the campaign was added to the strategic plan in the facilities. The introduction of the theme in medical undergraduate training and related radiology professions (e.g., radiography and nursing) could contribute to solidify the campaign as well as justify and optimise multidisciplinary actions [3, 8, 9].

The initial decrease on the number of the exams to 0–4 years old from May to July in the beginning of the campaign was related to the mobilisation of the campaign by web meetings in March and April engaging the multi professional team including a manager, radiographers, radiologists, and paediatricians. The increase in the number of exams of Chest X-ray and Paranasal Sinuses X-ray referrals, mainly to 0–4 years old, between the months May and July (wintertime in Brazil) may be due to respiratory diseases prevalence in this time, and between September and November is probably due to the period preceding the population's summer vacation. Most respiratory diseases are self-limited, not requiring radiological exams, showing the need for justification and awareness of physicians and parents/guardians [23, 29].

It observed, in general, many referrals for Chest X- Rays in the youngest age groups (53%). IAEA reinforces the importance to actualise physicians and radiologists about guidelines and clinical indications support.

Participants reported voluntarily, during the quarterly meetings, that the main causes of overused referrals for radiological procedures, such as difficulty in accessing medical records in the care sector, pressure from parents, lack of institutional protocols and defensive medicine, lack of knowledge of parents and professionals about ionising radiation effects, lack of time in medical care, some of these causes resonate with common reasons identified by the WHO [3]. Likewise, frequent emergency referrals such as, the use of tomography in minor head trauma without relevant clinical [1] and abdominal pain in children can be evaluated appropriately before requesting with the use of institutional recommendations [30, 31].

Representatives of the units reported that the parents were surprised by the information about the cumulative effect of radiation and accepted if the resolution of the clinical examination was explained to them. Larson reported that communicating the risk of the exam to the parents before the exam is positive for their understanding and did not cause a refusal to perform it if it is justified by the requesting physician [32]. In this project, we trained health professionals and receptionists to communicate with parents without causing alarm, emphasising that the decision is made by the requesting physician and that it has risks, and its use should be considered. The card and the educational instruments (banners, folders, videos at the reception) were important resources for understanding the risks of ionising radiation.

The involvement of the multidisciplinary team in this approach has been relevant since the reception, where the parents first reported that they came to perform the exam, as if it was a "photograph", without biological effects. Radiation protection campaign helped to clarify to physicians and patients the risks and benefits of radiological exams [10], and its instruments give credibility, facilitating adherence to the campaign [33]. The individual printed dose card and the educational instruments (banners, folders, videos) at the reception were important resources for raising awareness about the risks of ionising radiation. Even if some parents lose or forget the paper card, it impacts on showing the importance of the history for the doctor to take the action and avoid repeating tests already performed. Some facilities introduced electronic data archiving, which can be accessed with a specific password.

The facilities were asked to create Radiation protection committees as part of the campaign. They were created in 15 units, with more significant results than the others and were incorporated in each health care facility. The units with radiation protection committees were more engaged in the campaign, likely due to being more organised and prepared by the multi-professional team. It reinforced that communication is especially important to change the culture and promote good practice in medicine [3]. These committees were incorporated in the facility strategy management to be continued after the campaign and contribute on monitoring referrals and education activities. The Bonn Call for Action initiative by IAEA emphasises communication to patients and the population as an important action to reduce unnecessary exposure [9]. Similarly, guidelines are important to standardise the protocols in the institution giving more confidence to doctors and parents, as WHO and IAEA reinforce [3, 8, 9]. Imaging guidelines promote the doctors and the parent's greater knowledge of ionising radiation exam indications and reduce unjustified radiological exams, contributing to the correct request of the exams, reducing patient’s radiation and overuse [4, 14, 15, 30].

A pioneer radioprotection pilot project, applied in a private hospital in Brazil [34, 35], was used as a model for this study and distributed nearly seventeen thousand individual dose cards to those who underwent radiological procedures. Like results presented here, the pilot project resulted in a reduction of 22% of exams/attendance in X-ray and CT radiologic exams and of 29% in the referral of two or more X-rays for the same child.

This project encountered many challenges which deserve mentioning. Firstly, the scale of the project arising from the multicentre design, continental dimensions of the country and different organisational structures and cultures between units, required considerable planning and organisation with regular meetings and communication needed to maximise engagement. Secondly, the lack of national regulation on justification of radiological examinations coupled with the requisite of creating new local radiological protection committees, meant the project relied heavily on voluntary participation of motivated professionals. Finally, as with any such projects of scale, operating cost was not trivial, but was well compensated by savings in resources and radiation burden from the demonstrated reduction in imaging referrals.

Throughout the present study, we emphasised to imaging professionals that if the radiological examination is necessary, especially tomography, dose optimisation recommendations should be followed [36].

### Limitations

The limitations of the present study primarily included non-probabilistic sampling; and resourcing difficulties which led to the withdrawal of participants, who were unable to support the campaign. One motivation for implementing the campaign was their integration into quality programs. In the quarterly meetings or e-mail communication, no diagnostic errors were reported by the facilities, during the study period despite regular reminders. It is possible however that such incidents may have occurred but were either not detected or reported. Formal quantitative checking of a sample of medical records would have been useful to verify this, although due to resource constraints was not performed here.

To enable culture change the continuation of the project is important to keep it alive in the institution, professionals, and the population. The radiation risk and benefit communication, recommendations and the tools to the stakeholders clarified the purpose of the campaign and as they are close to patient's care, they are an important piece in the effectiveness of the process.

### Contributions for the area

The campaign improved the awareness that may impact on justification of imaging referrals for children aged 0–12 years, especially to the 0–4 years old group, which are the cohort with the most risk of radiation effects, contributing to the patient's security.

The study can be generalised to other emergency services if it follows the campaign methodology, its evaluation, and permanent education aiming at the continuity of the new behaviors.

## Conclusion

The campaign resulted in a substantial reduction in radiological referrals while promoting a radiation protection culture in the department. Simple education initiatives can contribute to both financial and radiation doses savings, particularly important in radiosensitive cohorts. Continuous education is especially important to change the culture of overuse, as well as information and communication to patients and carers about the benefits and risks of ionising radiation.

### Future

The group has done other initiatives in training and education with medical students and residents, reinforcing a National Radiation Protection Committee, and establishing Diagnostic Reference Level for CT exams in Brazil and is going to return to the justification campaign next year. It is recommended to continue to organise an annual meeting post campaign with the participants and future studies could investigate the ideal recommended period between campaigns. Further research including patients' perceptions on physician communications about radiological exams, as well as, about ionisation radiation exams awareness, would help to understand populations feelings, values, and knowledge about the theme, helping to create strategies to change the culture. A similar adult radiological protection campaign will be encouraged in the future in Brazil.

## Summary sentence

The project implemented a successful campaign (25% reduction in referrals of paediatric radiological exams) in the emergency department, through educational tools, radioprotection implementation guidelines and recommendations to improve patient’s safety and medical practice quality.

## Data Availability

All data relevant to the study are included in the article.

## Abbreviations

ACR: American college of radiology
ALARA: As low as reasonably achievable
CT: Computed tomography
ESR: European society of radiology
RCR: Royal college of radiologists
WHO: World Health Organization
